# ABT-126 monotherapy in mild-to-moderate Alzheimer’s dementia: randomized double-blind, placebo and active controlled adaptive trial and open-label extension

**DOI:** 10.1186/s13195-016-0210-1

**Published:** 2016-10-18

**Authors:** Laura M. Gault, Robert A. Lenz, Craig W. Ritchie, Andreas Meier, Ahmed A. Othman, Qi Tang, Scott Berry, Yili Pritchett, Weining Z. Robieson

**Affiliations:** 1 AbbVie, Inc., 1 North Waukegan Rd, North Chicago, IL 60064 USA; 2 Present Address: Amgen, Thousand Oaks, CA USA; 3 University of Edinburgh, Edinburgh, UK; 4 Present Address: Pfizer, Cambridge, MA USA; 5 Department of Pharmaceutics, Faculty of Pharmacy, Cairo University, Cairo, Egypt; 6 Berry Consultants, LLC, Austin, TX USA; 7 Present Address: MedImmune, Inc., Gaithersburg, MD USA

**Keywords:** ABT-126, Randomized controlled trial, Alzheimer’s disease, Assessment of cognitive disorders/dementia, Alzheimer’s dementia/drug therapy, Nicotinic agonists, Adaptive design

## Abstract

**Background:**

Results from a phase 2a study indicated that treatment with the novel α7 nicotinic acetylcholine receptor agonist ABT-126 25 mg once daily (QD) was associated with a trend for improvement in cognition in subjects with mild-to-moderate Alzheimer’s dementia (AD). A phase 2b program was designed to evaluate a broader dose range of ABT-126 as monotherapy in subjects with mild-to-moderate AD. The program consisted of a double-blind, placebo and active controlled study of ABT-126 (dose range 25–75 mg) and an open-label extension study (75 mg).

**Methods:**

The randomized double-blind study enrolled 438 subjects (Mini-Mental Status Examination score of 10–24, inclusive) not currently taking acetylcholinesterase inhibitors or memantine. Subjects received 24 weeks of ABT-126 25 mg QD (*n* = 77), ABT-126 50 mg QD (*n* = 108), ABT-126 75 mg QD (*n* = 73), donepezil 10 mg QD (*n* = 76), or placebo (*n* = 104). The primary endpoint was the change from baseline to week 24 in the 11-item Alzheimer's Disease Assessment Scale—Cognitive subscale (ADAS-Cog) total score. Subjects completing the double-blind study could enroll in the 28-week open-label extension study. Adverse events (AEs) and other safety parameters were monitored in both studies.

**Results:**

A total of 367 patients (83.8 %) completed the double-blind study and 349 (79.7 %) entered the open-label study. Compared with placebo, donepezil significantly improved ADAS-Cog 11-item total scores from baseline to week 24 (−2.29 ± 0.95; one-sided *P* = 0.008). No ABT-126 dose demonstrated a statistically significant improvement vs placebo at week 24 in the ADAS-Cog total score: ABT-126 25 mg, −0.47 ± 0.94 (*P* = 0.309); ABT-126 50 mg, −0.87 ± 0.85 (*P* = 0.153); and ABT-126 75 mg, −1.08 ± 0.94 (*P* = 0.127). Rates of serious AEs and discontinuations due to AEs were similar across treatment groups. The most frequently reported AEs in both studies were constipation, fall, and headache. No clinically meaningful changes were observed in other parameters.

**Conclusions:**

In the double-blind trial, donepezil significantly improved ADAS-Cog scores but no statistically significant improvement was seen with any ABT-126 dose. ABT-126 had an acceptable safety profile in subjects with mild-to-moderate AD in both studies.

**Trial registration:**

ClinicalTrials.gov NCT01527916, Registered 3 February 2012 (randomized trial). ClinicalTrials.gov NCT01676935. Registered 29 August 2012 (open-label extension study).

**Electronic supplementary material:**

The online version of this article (doi:10.1186/s13195-016-0210-1) contains supplementary material, which is available to authorized users.

## Background

Alzheimer’s dementia (AD) is a neurodegenerative disorder currently affecting nearly 5 million adults over the age of 65 years in the United States, a number that is expected to significantly increase over the next 40 years [1]. The few available marketed products, acetylcholinesterase inhibitors (AChEIs) and *N*-methyl-D-aspartate receptor antagonists, provide transient and modest improvements in cognition. Even when disease-modifying treatments are available to slow the course of the degenerative process, a need will remain for symptomatic treatments for patients with mild-to-moderate dementia.

A novel approach to symptomatic treatment for AD is modulation of the α7 nicotinic acetylcholine receptors (nAChRs), acetylcholine-gated cation channels found in areas of the brain that are important for learning and memory [2]. Activation of α7 nAChRs leads to an influx of calcium and activation of second messenger systems, and ultimately a release of a number of other neurotransmitters important for cognition [3]. The mechanism of action of α7 nAChR agonists is expected to differ from marketed compounds such as AChEIs that inhibit the enzyme which degrades acetylcholine in the synaptic cleft, resulting in an increased effect from acetylcholine at all receptor subtypes. By eliminating the dose-limiting toxicity associated with AChEIs acting at muscarinic and other nAChR subtypes that are not procognitive yet mediate untoward side effects [4], the efficacy and side-effect profiles of α7 nAChR agonists may differ from AChEIs [5]. Several α7 nAChR partial agonists have shown potential in the treatment of AD [6, 7] but either the procognitive effects were not reproducible [8] or the trials are currently on clinical hold, apparently due to safety concerns related to gastrointestinal side effects [9].

ABT-126 is a potent α7 nAChR agonist that displayed high affinity (Ki = 12–14 nM) at human, rat, and mouse α7 nAChRs in nonclinical studies. The in-vitro selectivity profile of ABT-126 was evaluated in a battery of radioligand binding assays (Cerep, France) that contained representatives of G-protein-coupled receptors and ligand/voltage-sensitive ion channels. In these assays, ABT-126 10 μM showed little binding, with the exception of the 5-HT3 receptor. ABT-126 had a Ki value of 140 nM (*n* = 6) at the 5-HT3 receptor, approximately 10-fold greater than the Ki value for displacement of α7 binding. In several animal models of cognition pertinent to AD, administration of ABT-126 resulted in signals of efficacy [10]. The safety and pharmacokinetic characteristics of ABT-126 were evaluated in single-dose and multiple-dose phase 1 studies that included healthy adults, healthy older subjects, clinically stable subjects with schizophrenia, and subjects with AD. Doses up to 150 mg once daily (QD) or up to 40 mg twice a day were generally well tolerated (unpublished data).

In a previously completed randomized, double-blind, phase 2a proof-of-concept study, 274 subjects with mild-to-moderate AD were treated for 12 weeks with 5 mg or 25 mg of ABT-126 QD, placebo, or donepezil [11]. The change from baseline to the final evaluation in the 11-item Alzheimer’s Disease Assessment Scale—Cognitive subscale (ADAS-Cog) [12, 13] total score was the primary endpoint. The ABT-126 25 mg dose showed some procognitive effect (ADAS-Cog least squares (LS) mean (standard error) difference from placebo −1.19 (0.90); one-sided *P* = 0.095) which was numerically less effective than that seen with donepezil treatment (−1.43 (0.90); one-sided *P* = 0.057). The 5 mg dose of ABT-126 had an effect similar to placebo. There was no imbalance in adverse events (AEs) across treatment groups or other safety signals that would preclude further investigation.

A plateau of ABT-126 efficacy could not be characterized based on the exposure–response analysis of the phase 2a study with a limited dose range of 5–25 mg, suggesting that testing of higher ABT-126 doses was needed to definitively characterize the procognitive potential. Additional toxicology and phase 1 data enabled testing higher doses. Therefore, this 24-week, randomized, double-blind, controlled phase 2b study was designed to evaluate a higher dose range of ABT-126 25 mg, 50 mg, and 75 mg QD in subjects with mild-to-moderate AD.

An adaptive design was chosen to achieve study objectives using a smaller sample size than a more traditional parallel group design. The study consisted of two parts, each with its own objectives. The objective of Part 1 was to efficiently characterize the dose–response relationship of ABT-126 using a response-adaptive randomization methodology. The objective of Part 2 was to provide additional safety data and efficacy data for less sensitive outcome measures (e.g., quality of life measurements) for the dose selected in Part 1. This efficient design had adequate power to characterize the dose–response relationship on the primary outcome measures (ADAS-Cog) with a smaller sample size in Part 1. Results from the double-blind 24-week trial and its 28-week open-label extension study are reported.

## Methods

### Subjects

Eligible subjects were 55–90 years of age and diagnosed with mild-to-moderate AD, defined as meeting National Institute of Neurological and Communicative Disorders and Stroke/Alzheimer’s Disease and Related Disorders Association (NINCDS/ADRDA) criteria for probable AD. Other key inclusion criteria were a Mini-Mental Status Examination (MMSE) [14] score of 10–24 (inclusive), a Cornell Scale for Depression in Dementia (CSDD) [15] score ≤ 10, and a Modified Hachinski Ischemic Scale (MHIS) [16] total score ≤ 4 at screening. Subjects who were currently receiving medication for AD or had taken such agents within 60 days of the first screening visit were excluded.

The protocol and informed consent were approved by institutional review boards or independent ethics committees (Additional file 1). Written informed consent was obtained from each subject and caregiver prior to study participation.

### Study design and treatment

#### Double-blind study

This was a randomized, double-blind, placebo and active controlled phase 2b study conducted to evaluate a range of ABT-126 doses as monotherapy in subjects with mild-to-moderate AD. The study consisted of two parts, each 24 weeks in duration with its own goals and randomization scheme. Part 1 of the study was designed to investigate the dose–response relationship for three doses of ABT-126 on the primary endpoint, the 11-item ADAS-Cog total score. A donepezil arm was included in Part 1 to permit comparisons of treatment effects of ABT-126 with a positive control and, ultimately, to verify assay sensitivity of the design and conduct of the study. The selected dose from Part 1 was continued into Part 2 to provide additional safety and efficacy data on other less sensitive outcome measures.

In Part 1, subjects were randomized to placebo, ABT-126 25 mg, ABT-126 50 mg, ABT-126 75 mg, or donepezil, identical in appearance. The first 100 subjects were randomized with equal probability to the five treatment arms. Subsequently, the randomization ratio for the three ABT-126 dose groups changed using a response-adaptive randomization design in which interim efficacy data were utilized to change ABT-126 treatment allocation probabilities from the initial 1:1:1 to favor the more efficacious ABT-126 dose group(s). A detailed description of the adaptive randomization is provided in Additional file 2.

For Part 2, the Data Monitoring Committee (DMC) selected a dose for further evaluation based on the efficacy and safety profile displayed in Part 1. In Part 2 subjects were randomized 1:1 to the selected ABT-126 dose or placebo. This allowed for a larger sample size for the selected dose group and the placebo group to evaluate the effect on secondary measures such as the Clinician Interview-Based Impression of Change (CIBIC) and Alzheimer’s Disease Cooperative Study—Activities of Daily Living (ADCS-ADL) that were expected to have lower effect sizes than the ADAS-Cog. Part 2 randomization was to continue until 100 subjects (total from Parts 1 and 2) were randomized to the selected ABT-126 dose group. In both parts, subjects were assigned to treatment using an interactive voice response/interactive Web-based system.

### Assessments in the double-blind study

#### Efficacy

In the double-blind study, baseline was defined as the last assessment taken on or before the day −1 study visit. The 11-item ADAS-Cog was assessed at weeks 4, 8, 12, 18, and 24. The primary efficacy endpoint was the 11-item ADAS-Cog at week 24. The ADAS-Cog 13-item total score was analyzed as a secondary efficacy variable. Other secondary efficacy measures were as follows: MMSE [14], CIBIC-Plus [17], Neuropsychiatric Inventory (NPI) [18, 19], ADCS-ADL [20], Wechsler Memory Scale—III (WMS-III) Working Memory Index [21], DEMentia Quality of Life (DEMQOL) [22], Partner–Patient Questionnaire for Shared Activities (PPQSA) [23], Resource Use in Dementia (RUD-Lite) [24], EuroQol-5D-5 L (EQ-5D-5 L) [25], and EuroQol-5D-3 L (EQ-5D-3 L) proxy. The timing of secondary efficacy assessments in the double-blind study is provided in Additional file 3.

#### Safety

In the double-blind study, the safety of ABT-126 was assessed through AE monitoring, concomitant medication review, vital signs, electrocardiograms (ECGs), physical examinations, brief neurological examinations, brief psychiatric assessments, Columbia-Suicide Severity Rating Scale (C-SSRS) [26], and clinical laboratory tests at each study visit and the CSDD at baseline and at the week 24/final visit. Telephone contacts conducted during weeks 6, 10, 15, and 21 and follow-up assessed AEs and concomitant medications. In addition, an independent DMC reviewed safety data throughout the double-blind trial.

#### Pharmacokinetic

A pharmacokinetic sample was obtained at each of the week 2, 4, 8, 12, 18, and 24/early discontinuation study visits. The dates and times of the previous dose of study drug and sample collection were recorded. ABT-126 plasma concentrations were determined using a validated liquid chromatography method with mass spectrometric detection at AbbVie (North Chicago, IL, USA).

#### Medication compliance

Pill counts were used to assess compliance with study drug at each study visit during the treatment period. Subjects taking ≥70 % of the study drug were considered compliant.

### Statistical analysis

All randomized subjects from Parts 1 and 2 who took ≥1 dose of study drug were combined and included in the intent-to-treat (ITT) and safety datasets. Prespecified one-sided tests at the significance level of 0.050 were utilized to evaluate the treatment effect for each ABT-126 dose group compared with placebo because ABT-126 had to demonstrate improvement over placebo to be considered effective. For the selected dose group and placebo, data from Parts 1 and 2 were combined to perform statistical analyses without multiplicity adjustment given the fact that this was a phase 2 study with data collected to inform the development plan for ABT-126.

Efficacy analyses were conducted using the ITT dataset and safety analyses on the safety dataset unless otherwise noted. The change from baseline to week 24 on the ADAS-Cog 11-item total score was the primary efficacy variable. The primary efficacy analysis used a likelihood-based, mixed-effects model for repeated measures (MMRM) that included fixed, categorical effects for treatment, site, visit, and treatment-by-visit interaction, with continuous fixed covariates for baseline score and the baseline score-by-visit interaction.

The ADAS-Cog 13-item total score, MMSE, NPI, ADCS-ADL, WMS-III Working Memory Index, DEMQOL, and PPQSA were analyzed by MMRM using the model described for the ADAS-Cog 11-item total score. Changes from baseline to final observation for variables RUD-Lite, EQ-5D-5 L, and EQ-5D-3 L proxy were analyzed by ANCOVA models with treatment and study site as the main effects and the baseline value as a covariate. Postbaseline CIBIC-Plus observations were analyzed by MMRM with the fixed effects of treatment, study site, visit, and treatment-by-visit interaction, with the Clinician Interview-Based Impression of Severity (CIBIS) score collected at day –1 as a covariate.

No adjustment for multiple comparisons was made for this phase 2 study with the primary objective being evaluating each ABT-126 dose relative to placebo. Subgroup analyses were also conducted as described in Additional file 4.

Analyses of treatment differences between each ABT-126 dose group and placebo as well as between donepezil and placebo were conducted for laboratory measurements, vital sign parameters, and ECG variables using an ANOVA with treatment as the factor. Statistical significance for safety variables was judged at a two-sided test significance level of 0.05.

#### Open-label extension study

Medically stable subjects could enroll in the extension study if they completed the double-blind study. Subjects were treated with ABT-126 75 mg QD for up to 28 weeks, with dosing reductions of 25 mg increments allowed for safety or tolerability reasons. Study visits occurred on day –1 and weeks 2, 4, 8, 16, and 28 with telephone contacts at weeks 12, 20, and 24 and follow-up (30 days after the last dose of study drug). The safety and tolerability of ABT-126 was evaluated by AE monitoring, concomitant medication review, vital signs, ECGs, physical examinations, brief neurological examinations, brief psychiatric assessments, C-SSRS, and clinical laboratory tests at each study visit and the CSDD at baseline and the week 28/final visit. Telephone contacts assessed AEs and concomitant medications.

## Results

### Subjects

The study was conducted at 33 sites from February 2012 through December 2013. A total of 557 subjects were screened and 438 subjects (78.6 %) were randomized in Russia (*n* = 153), South Africa (*n* = 95), the United Kingdom (*n* = 65), Ukraine (*n* = 50), Poland (*n* = 39), and the United States (*n* = 36). Two randomized subjects did not take the study drug.

Interim adaptation of randomization began after 100 subjects had been randomized. Randomization probabilities were updated every 2 weeks based on cumulative efficacy information throughout the study (Additional file 2). At the final analysis in Part 1, subjects were distributed across treatment dose groups as follows: 60 placebo, 77 ABT-126 25 mg, 64 ABT-126 50 mg, 73 ABT-126 75 mg, and 76 donepezil. When 330 subjects were enrolled, the Efficacy DMC reviewed the interim results, including simulation data, effect size calculations, and safety/tolerability information from the Safety DMC, and recommended that the trial proceed to Part 2 using ABT-126 50 mg. In Part 2, 88 additional subjects were enrolled, 40 to ABT-126 50 mg and 48 to placebo, resulting in a total of 104 subjects in the placebo group and 108 in the 50 mg group.

Demographic and baseline characteristics are presented in Table 1. No statistically significant differences were observed among treatment groups. Approximately 30 % of subjects had taken drugs for AD before the entering the study, most frequently AD medications in the “Other” category (14.7 %; e.g., piracetam, bapineuzemab, and other investigational agents), donepezil (7.1 %), galantamine (6.9 %), and memantine (6.0 %).

**Table 1:** Baseline demographic characteristics in the double-blind controlled study

	Placebo	ABT-126 25 mg	ABT-126 50 mg	ABT-126 75 mg	Donepezil 10 mg	All subjects
Number of subjects	104	77	107	73	75	436
Age (years), mean ± SD	73.2 ± 7.39	73.0 ± 7.62	73.9 ± 8.26	76.2 ± 8.14	75.1 ± 7.75	74.2 ± 7.89
Age < 75 years, *n* (%)	59 (56.7)	37 (48.1)	50 (46.7)	30 (41.1)	30 (40.0)	206 (47.2)
Age ≥ 75 years, *n* (%)	45 (43.3)	40 (51.9)	57 (53.3)	43 (58.9)	45 (60.0)	230 (52.8)
Female, *n* (%)	65 (62.5)	40 (51.9)	68 (63.6)	52 (71.2)	40 (53.3)	265 (60.8)
Male, *n* (%)	39 (37.5)	37 (48.1)	39 (36.4)	21 (28.8)	35 (46.7)	171 (39.2)
White, *n* (%)	91 (87.5)	75 (97.4)	96 (89.7)	64 (87.7)	70 (93.3)	396 (90.8)
BMI (kg/m^2^), mean ± SD	26.2 ± 3.88	27.1 ± 4.91	26.0 ± 4.98	25.5 ± 4.62	25.0 ± 4.27	26.0 ± 4.57
Age at AD symptom onset (years), mean ± SD	69.6 ± 7.66	69.2 ± 8.51	70.2 ± 8.67	72.3 ± 7.97	71.2 ± 8.24	70.4 ± 8.25
Years since AD symptom onset,^a^ mean ± SD	3.7 ± 2.61	4.1 ± 2.96	3.9 ± 3.11	4.2 ± 2.67	4.2 ± 2.45	4.0 ± 2.78
Age at AD diagnosis (years), mean ± SD	72.1 ± 7.60	71.5 ± 8.10	72.5 ± 8.64	74.8 ± 8.30	73.6 ± 7.91	72.8 ± 8.16
Years since AD diagnosis, mean ± SD	1.1 ± 1.79	1.5 ± 1.91	1.4 ± 2.37	1.5 ± 1.96	1.6 ± 1.92	1.4 ± 2.02
Family history of AD, *n* (%)	22 (21.2)	20 (26.0)	23 (21.5)	6 (8.2 %)	10 (13.3)	81 (18.6)
ADAS-Cog (11-item),^b^ mean ± SD	26.1 ± 10.98	24.6 ± 11.45	25.6 ± 11.32	27.2 ± 9.81	27.9 ± 12.08	26.2 ± 11.16
MMSE score, mean ± SD	19.1 ± 4.00	20.0 ± 4.09	18.6 ± 4.03	18.6 ± 3.87	18.4 ± 4.42	18.9 ± 4.09
Number of subjects	92	66	97	63	64	382
APOE ε4 positive, *n* (%)	37 (40.2)	34 (51.5)	48 (49.5)	27 (42.9)	32 (50.0)	178 (46.6)

^a^Time from onset of AD symptoms or diagnosis to first dose of study drug

^b^Baseline results based on a total of 435 subjects

*AD* Alzheimer’s dementia, *ADAS-Cog*, Alzheimer’s Disease Assessment Scale-Cognitive subscale, *APOE* apolipoprotein E, *BMI* body mass index, *MMSE* Mini-Mental Status Examination, *SD* standard deviation

**Fig. 1:**
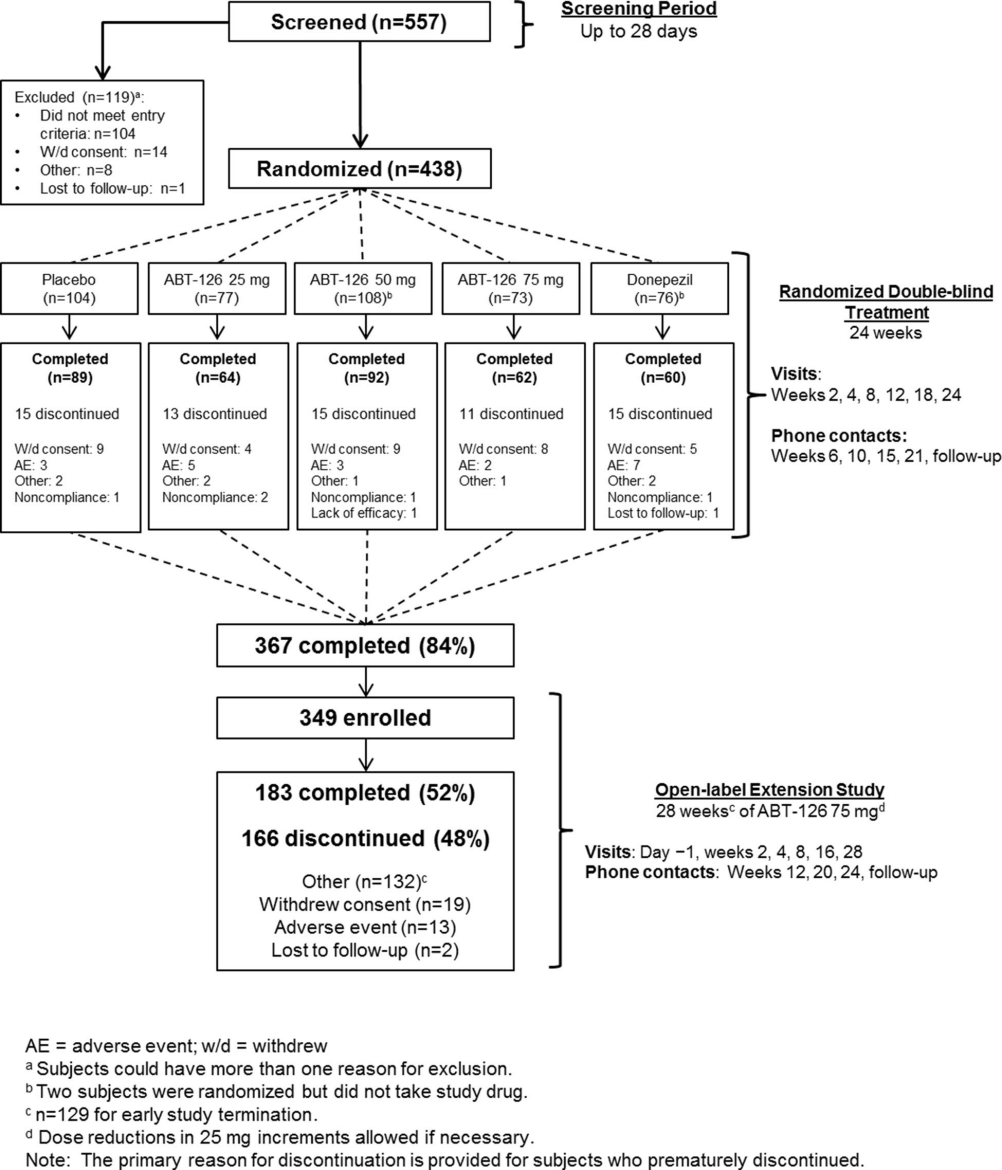
Study design and subject disposition

Subject disposition is presented in Fig. 1. A total of 367 subjects (84.2 %) completed the double-blind study and 69 subjects (15.8 %) discontinued prematurely. Rates of early discontinuation were similar across treatment groups. The most frequently reported reasons for leaving the study were withdrawn consent (*n* = 35, 8.0 %) and AEs (*n* = 20, 4.6 %).

### Efficacy

**Fig. 2:**
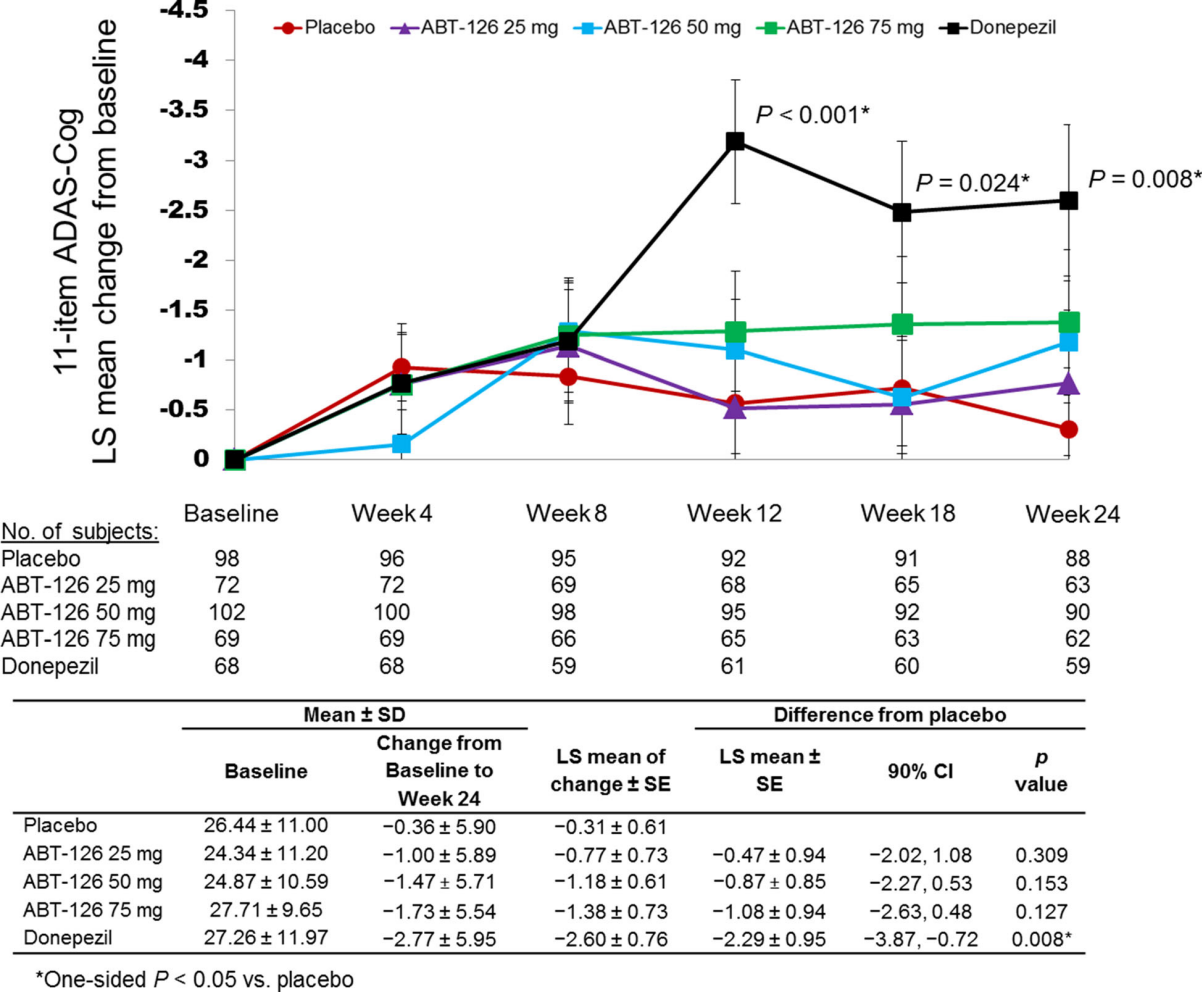
LS mean change from baseline over time in 11-item ADAS-Cog total score. Maximum likelihood, mixed-effect, repeated-measures analysis of change from baseline at each study visit for the ADAS Cog 11-item total score (ITT dataset). Standard error of the LS means represented by *error bars. ADAS-Cog* Alzheimer’s Disease Assessment Scale—Cognitive subscale, *LS* least squares

Twenty-nine subjects were not included in the primary efficacy analysis because they did not have a baseline or ontreatment ADAS-Cog 11-item total score (*n* = 27) or they took no study drug (*n* = 2). No statistically significant improvement over placebo from baseline to week 24 in ADAS-Cog 11-item total score was observed for any of the ABT-126 treatment groups (Fig. 2). LS mean differences from placebo at week 24 were –0.47 for ABT-126 25 mg (90 % CI −2.02, 1.08; one-sided *P* = 0.309), –0.87 for ABT-126 50 mg (90 % CI −2.27, 0.53; *P* = 0.153), and –1.08 for ABT-126 75 mg (90 % CI −2.63, 0.48; *P* = 0.127). The donepezil group demonstrated statistically significant improvement compared with placebo from baseline to week 24 in the ADAS-Cog 11-item total score (LS mean difference from placebo −2.29, 90 % CI −3.87, −0.72; *P* = 0.008).

A prespecified Bayesian dose–response analysis of the change from baseline to final analysis in the ADAS-Cog 11-item total score using the Part 1 ITT dataset indicated that ABT-126 50 mg and ABT-126 75 mg were more efficacious than ABT-126 25 mg but less effective than donepezil. Changes from baseline to week 24 for the ADAS-Cog 11-item total score from the Part 1 ITT dataset were also analyzed using an MMRM similar to that used in the primary analysis. Findings were consistent with the Bayesian dose–response analysis.

MMRM results for the change from baseline to week 24 for secondary efficacy measures are presented in Table 2. Results from an MMRM analysis of the ADAS-Cog 13-item total score change from baseline to each study visit were consistent with the ADAS-Cog 11-item scale results, because no dose of ABT-126 demonstrated a statistically significant improvement compared with placebo. Secondary efficacy analyses of the ADAS-Cog 13-item and ADAS-Cog (Items 1, 4, and 8) total scores were consistent with those observed with the ADAS-Cog 11-item scale.

**Table 2:** Double-blind secondary efficacy: change from baseline to final analysis from repeated-measures analyses

	Baseline		Week 24, *n*	Change from BL to week 24		Difference from placebo		
	*n*	Observed mean (SD)		Mean (SD)	LS mean ± SE	LS mean ± SE	90 % CI	*P* value
Alzheimer’s Disease Assessment Scale—Cognitive subscale, 13-item total score (↓ indicates improvement)								
Placebo	98	38.26 (12.66)	88	−0.76 (6.99)	−0.67 ± 0.71			
ABT-126 25 mg	71	35.05 (13.06)	63	−1.41 (6.82)	−1.14 ± 0.84	−0.47 ± 1.08	−2.25, 1.31	0.333
ABT-126 50 mg	100	36.71 (12.52)	87	−2.12 (6.41)	−1.70 ± 0.71	−1.03 ± 0.98	−2.65, 0.60	0.149
ABT-126 75 mg	68	39.11 (10.72)	61	−2.32 (6.07)	−1.87 ± 0.84	−1.19 ± 1.09	−2.98, 0.60	0.137
Donepezil	68	39.18 (14.13)	59	−3.56 (6.69)	−3.54 ± 0.87	−2.86 ± 1.10	−4.67, −1.05	0.005*
Mini-Mental Status Examination score (↑ indicates improvement)								
Placebo	98	18.97 (4.01)	89	0.56 (2.83)	0.39 ± 0.32			
ABT-126 25 mg	72	20.08 (4.04)	64	0.19 (3.13)	−0.16 ± 0.38	−0.55 ± 0.49	−1.36, 0.27	0.866
ABT-126 50 mg	102	18.70 (3.92)	92	0.86 (3.38)	0.78 ± 0.32	0.39 ± 0.44	−0.34, 1.12	0.191
ABT-126 75 mg	69	18.39 (3.88)	62	0.74 (3.17)	0.53 ± 0.38	0.14 ± 0.49	−0.68, 0.95	0.390
Donepezil	69	18.59 (4.42)	59	1.29 (2.76)	1.19 ± 0.39	0.80 ± 0.50	−0.02, 1.62	0.055
Clinician Interview-Based Impression of Severity and Clinician Interview-Based Impression of Change—Plus^a^								
Placebo	98	3.64 (0.79)	89	4.15 (0.83)	4.18 ± 0.09			
ABT-126 25 mg	73	3.77 (0.86)	64	4.03 (0.73)	4.06 ± 0.10	−0.12 ± 0.13	−0.33, 0.10	0.184
ABT-126 50 mg	102	3.63 (0.87)	92	3.95 (0.87)	4.03 ± 0.08	−0.15 ± 0.12	−0.34, 0.05	0.105
ABT-126 75 mg	68	3.75 (0.82)	61	3.77 (0.84)	3.80 ± 0.10	−0.38 ± 0.13	−0.59, −0.16	0.002*
Donepezil	69	3.88 (0.81)	59	3.68 (0.78)	3.75 ± 0.10	−0.43 ± 0.13	−0.65, −0.21	<0.001*
Neuropsychiatric Inventory, 10-item total score (↓ indicates improvement)								
Placebo	98	8.64 (8.70)	89	0.18 (6.27)	−0.26 ± 0.82			
ABT-126 25 mg	73	9.21 (8.93)	64	−1.72 (7.81)	−1.09 ± 0.96	−0.82 ± 1.23	−2.86, 1.21	0.252
ABT-126 50 mg	102	8.30 (8.07)	93	−0.59 (9.78)	−0.50 ± 0.81	−0.24 ± 1.11	−2.07, 1.60	0.416
ABT-126 75 mg	69	10.57 (11.31)	62	−0.98 (7.26)	−0.13 ± 0.96	0.14 ± 1.25	−1.92, 2.19	0.544
Donepezil	69	12.39 (11.63)	59	−3.27 (9.87)	−2.72 ± 0.99	−2.45 ± 1.26	−4.53, −0.38	0.026*
Neuropsychiatric Inventory, 12-item total score (↓ indicates improvement)								
Placebo	98	10.17 (9.66)	89	0.30 (7.67)	−0.11 ± 0.95			
ABT-126 25 mg	73	11.19 (10.45)	64	−1.91 (9.84)	−0.92 ± 1.11	−0.81 ± 1.44	−3.17, 1.56	0.287
ABT-126 50 mg	102	9.43 (8.55)	93	−0.03 (11.12)	0.05 ± 0.94	0.16 ± 1.30	−1.98, 2.30	0.549
ABT-126 75 mg	69	12.19 (12.93)	62	−1.52 (8.01)	−0.54 ± 1.12	−0.43 ± 1.45	−2.82, 1.96	0.383
Donepezil	69	13.41 (12.77)	59	−3.10 (10.82)	−2.67 ± 1.15	−2.55 ± 1.46	−4.96, −0.15	0.041*
Alzheimer’s Disease Cooperative Study—Activity of Daily Living total score (↑ indicates improvement)								
Placebo	98	56.46 (14.17)	89	−1.79 (8.16)	−2.30 ± 0.76			
ABT-126 25 mg	73	57.15 (16.35)	64	0.28 (6.65)	−0.44 ± 0.89	1.86 ± 1.14	−0.03, 3.74	0.053
ABT-126 50 mg	102	55.97 (14.96)	93	0.18 (7.13)	0.00 ± 0.75	2.30 ± 1.04	0.60, 4.01	0.013*
ABT-126 75 mg	69	54.30 (14.65)	62	−0.21 (7.18)	−0.44 ± 0.90	1.86 ± 1.15	−0.04, 3.76	0.054
Donepezil	69	52.00 (14.11)	59	2.14 (5.60)	1.71 ± 0.92	4.01 ± 1.16	2.09, 5.93	<0.001*
	Baseline		Week 18, *n*	Change from BL to week 18		Difference from placebo		
	*n*	Observed mean (SD)		Mean (SD)	LS mean ± SE	LS mean ± SE	90 % CI	*P* value
Wechsler Memory Scale—III Working Memory Index total score (↑ indicates improvement)								
Placebo	93	74.02 (13.28)	91	2.04 (11.21)	1.17 ± 1.11			
ABT-126 25 mg	68	78.50 (17.02)	65	0.83 (11.38)	0.24 ± 1.31	−0.93 ± 1.64	−3.63, 1.77	0.715
ABT-126 50 mg	99	75.57 (13.40)	93	−0.42 (11.32)	−1.73 ± 1.10	−2.91 ± 1.48	−5.34, −0.47	0.975
ABT-126 75 mg	66	77.85 (15.40)	64	−1.66 (10.66)	−2.34 ± 1.27	−3.52 ± 1.65	−6.23, −0.80	0.983
Donepezil	61	75.66 (15.54)	61	1.82 (8.88)	1.31 ± 1.35	0.13 ± 1.65	−2.60, 2.86	0.468

^*^One-sided *P* value statistically significant vs placebo

^a^Clinician Interview-Based Impression of Severity at baseline and Clinician Interview-Based Impression of Change—Plus at subsequent visits. Clinician Interview-Based Impression of Change—Plus ratings range from 1 = markedly improved to 7 = markedly worse. LS means (SE) are presented instead of LS mean (SE) of change

*BL* baseline (last assessment taken on or before the day –1 visit), *LS* least squares, *SD* standard deviation, *SE* standard error

Statistically significant improvements in the LS mean difference from placebo at week 24 were seen on the CIBIC-Plus for ABT-126 75 mg (–0.38, 90 % CI −0.59, −0.16; *P* = 0.002) and for donepezil (–0.43, 90 % CI −0.65, −0.21; *P* < 0.001). A Cochran–Mantel–Haenszel analysis of the CIBIC-Plus also showed significant treatment effects for ABT-126 75 mg (*P* = 0.009) and donepezil (*P* = 0.003) at week 24.

Significant improvements in the LS mean difference compared with placebo at week 24 were seen on the ADCS-ADL total score for ABT-126 50 mg (2.30, 90 % CI 0.60, 4.01; *P* = 0.013) and donepezil (4.01, 90 % CI 2.09, 5.93; *P* < 0.001); trends were observed for both ABT-126 25 mg and ABT-126 75 mg (*P* = 0.053 and *P* = 0.054, respectively). Compared with placebo, the basic ADCS-ADL total score had statistically significant differences for all treatment groups at week 24 (all *P* < 0.05) and the instrumental ADCS-ADL total score showed statistically significant differences for ABT-126 50 mg (*P* = 0.039) and donepezil (*P* = 0.001) at week 24.

Using the ANCOVA model, statistically significant differences for all treatment groups at the final evaluation were seen in RUD-Lite caregiver time compared with placebo, but these differences were attributed to outlier values in the placebo group that exceeded the defined maximum number of 720 reportable caregiver hours (range: –206 to 2347 hours). No statistically significant differences were observed at the final evaluation in other secondary measures (MMSE, NPI, WMS-III, DEMQOL, PPQSA, EQ-5D-5 L, and EQ-5D-3 L proxy) when ABT-126 dose groups were compared with placebo. Results from subgroup analyses demonstrated some modest treatment effects for ABT-126 that did not surpass the magnitude of effect shown by donepezil. Statistically significant treatment by subgroup interactions for country (*P* = 0.005), baseline age category (*P* = 0.041), and baseline MMSE category (*P* = 0.006) were observed in ADAS-Cog 11-item total score subgroup analyses (Additional file 4).

### Safety

AEs are summarized in Table 3. Of the 436 randomized and treated subjects, 245 (56.2 %) had at least one AE. The rates of reported AEs were generally similar among treatment groups (Table 3). Most AEs (95.5 %) were assessed by the investigator as mild or moderate in severity. Urinary tract infection was the only severe AE experienced by more than one subject (one subject receiving ABT-126 50 mg and one subject receiving placebo).

**Table 3:** Summary of treatment-emergent adverse events from both studies, *n* (%)

	Double-blind study					Open-label study
	Placebo (*n* = 104)	ABT-126 25 mg (*n* = 77)	ABT-126 50 mg (*n* = 107)	ABT-126 75 mg (*n* = 73)	Donepezil 10 mg (*n* = 75)	ABT-126 75 mg (*n* = 349)
Any AE	56 (53.8)	42 (54.5)	62 (57.9)	38 (52.1)	47 (62.7)	167 (47.9)
Possibly related^a^	20 (19.2)	16 (20.8)	27 (25.2)	14 (19.2)	24 (32.0)	66 (18.9)
Discontinued due to AE	4 (3.8)	5 (6.5)	7 (6.5)	2 (2.7)	9 (12.0)	13 (3.7)
Severe AE	2 (1.9)	3 (3.9)	2 (1.9)	1 (1.4)	3 (4.0)	18 (5.2)
Serious AE	5 (4.8)	6 (7.8)	7 (6.5)	2 (2.7)	3 (4.0)	17 (4.9)
Deaths^b^	0	0	0	0	2 (2.7)^c^	4 (1.1)^d^
AEs reported by ≥ 5.0 % of subjects in any treatment group in the double-blind study (MedDRA preferred term)						
Constipation^e^	3 (2.9)	7 (9.1)	16 (15.0)^e^	3 (4.1)	2 (2.7)	17 (4.9)
Headache	7 (6.7)	5 (6.5)	4 (3.7)	5 (6.8)	8 (10.7)	11 (3.2)
Fall	4 (3.8)	4 (5.2)	5 (4.7)	3 (4.1)	5 (6.7)	15 (4.3)
Nausea	3 (2.9)	1 (1.3)	4 (3.7)	2 (2.7)	6 (8.0)	7 (2.0)
Diarrhea	2 (1.9)	2 (2.6)	4 (3.7)	2 (2.7)	4 (5.3)	6 (1.7)
Anxiety	1 (1.0)	1 (1.3)	7 (6.5)	2 (2.7)	0	6 (1.7)
Depressed mood	1 (1.0)	1 (1.3)	0	0	4 (5.3)	3 (0.9)

^a^Any AE determined by the investigator as having a reasonable possibility of being related to the study drug

^b^Includes all deaths, whether or not considered treatment emergent. In both studies all events related to deaths were considered as having no reasonable possibility of being related to the study drug

^c^Cerebrovascular accident (day 6) and septic shock (day 143)

^d^Advanced age (open-label extension day 7); cerebral infarction, brain edema, brain stem syndrome, and pulmonary edema (open-label extension day 40); arterial thrombosis and gastrointestinal necrosis (open-label extension day 21); and myocardial infarction (open-label extension day 172)

^e^Statistically significant vs placebo (*P* = 0.0019 for ABT-126 combined and *P* = 0.003 for ABT-126 50 mg)

All data are presented as *n* (%)

*AE* adverse event, *MedDRA* Medical Dictionary for Regulatory Activities, *n* number of subjects

Constipation occurred in a significantly higher proportion of subjects administered ABT-126 (10.1 % combined and 15.0 % for 50 mg) compared with placebo (2.9 %; *P* = 0.019 and *P* = 0.003, respectively). Of the 26 subjects taking ABT-126 who had an AE of constipation, 17 cases (65.4 %) were considered mild, eight cases (30.8 %) were moderate, and one case (3.8 %) was severe.

Twenty-seven subjects (6.2 %) discontinued from the study prematurely because of an AE (Table 3). Constipation was the only AE leading to discontinuation in more than one subject taking ABT-126 (one subject receiving ABT-126 25 mg and two subjects receiving ABT-126 50 mg). Significantly more subjects in the donepezil group discontinued prematurely for any AE compared with placebo (*P* = 0.045). This finding appeared to be driven by three donepezil-treated subjects (4.0 %) who left the study due to gastrointestinal disorders.

The frequency of serious adverse events (SAEs) was similar across treatment groups, occurring in 23 subjects (5.3 %) overall (Table 3). Constipation and urinary tract infection were the only SAEs that occurred in more than one subject taking ABT-126 (two subjects each). Two deaths occurred during the study, both in the donepezil group. One subject experienced a cerebrovascular accident on day 3, was hospitalized, and died 3 days later. Another subject died of septic shock on day 143, 1 day after being hospitalized for cholelithiasis. Both events were judged by the investigator as having no reasonable possibility of being related to the study drug.

No consistent clinically meaningful mean changes or dose-related trends were detected in laboratory, vital signs, ECG findings, CSDD total scores, and C-SSRS for any group in the double-blind study.

### Pharmacokinetic

ABT-126 concentrations were consistent with the expected plasma concentrations based on previous pharmacokinetic assessments in healthy volunteers and subjects with AD. In this trial, the percentage of subjects with consistently (on three or more visits) low plasma concentrations (below the minimum simulated for each dose level) was approximately 4 % across the ABT-126 dose levels, suggesting that a high percentage of the subjects (approximately 96 %) had reasonable compliance with the study drug.

### Medication compliance

Overall 93.8 % of subjects in the double-blind study were considered by the investigator to be compliant with the study drug at least 70 % of the time. A significantly smaller proportion of subjects taking donepezil were treatment compliant at week 4 (92.8 %; *P* = 0.005) compared with the other treatment groups (ABT-126 25 mg, 100 %; ABT-126 50 mg, 99.0 %; ABT-126 75 mg, 98.5 %; placebo, 100 %); this difference was not observed in subsequent weeks.

### Open-label extension study

A total of 349/367 subjects (95.1 %) who completed the double-blind study enrolled into the open-label extension study (62 ABT-126 25 mg, 86 ABT-126 50 mg, 57 ABT-126 75 mg, 87 placebo, 57 donepezil). The open-label extension study was terminated early following completion of the double-blind study because ABT-126 did not demonstrate adequate efficacy in two randomized phase 2 studies. No efficacy analyses were conducted. A total of 183 subjects (52.4 %) completed the study and 166 subjects (47.6 %) discontinued the study prematurely—129 discontinuations (77.7 %) were due to the termination of the study (Fig. 1). Thirteen subjects (3.7 %) discontinued prematurely due to an AE.

Participating in the open-label study were 210 females (60.2 %) and 139 males (39.8 %), with a mean age of 74.1 years; 90.0 % were white. The baseline mean (SD) MMSE total score was 19.7 (5.00). All subjects took at least one dose of ABT-126 75 mg. Twelve subjects (3.4 %) had decreased to ABT-126 50 mg QD, and one subject increased back to ABT-126 75 mg QD.

During the open-label study 167 subjects (47.9 %) reported at least one AE. The most frequently reported AEs were constipation (4.9 %), fall (4.3 %), headache (3.2 %), agitation (2.3 %), irritability (2.3 %), and nausea (2.0 %; Table 3). Approximately 90 % of the AEs were considered mild or moderate in severity, and 18 subjects (10.8 %) experienced a severe AE. A total of 66 subjects (18.9 %) had an AE assessed by the investigator as having a reasonable possibility of being related to the study drug.

SAEs were experienced by 17 subjects (4.9 %). Convulsion (three subjects, 0.9 %) and femoral neck fracture (two subjects, 0.6 %) were the only SAEs reported by ≥ 2 subjects. One SAE of convulsion was considered as having a reasonable possibility of being related to the study drug.

There were four deaths during the study: myocardial infarction (open-label extension day 172); advanced age (open-label extension day 7); cerebral infarction, brain edema, brain stem syndrome, and pulmonary edema (open-label extension day 40); and arterial thrombosis and gastrointestinal necrosis (open-label extension day 21). All events leading to the deaths were considered by the investigator as having no reasonable possibility of being related to the study drug. Similar to the double-blind study, no clinically significant trends were identified in laboratory results, vital signs, ECG findings, CSDD total scores, and C-SSRS.

## Discussion

In this randomized double-blind trial of multiple doses of ABT-126, none of the ABT-126 groups had statistically significant improvement compared with placebo from baseline to week 24 on the ADAS-Cog 11-item total score. The ABT-126 50 mg and ABT-126 75 mg dose groups showed statistically significant improvements over placebo on the ADCS-ADL and CIBIC-Plus, respectively, but the improvement was numerically lower than that observed with donepezil. Similarly, some subgroup analyses showed a statistically significant improvement over placebo on the ADAS-Cog (e.g., MMSE < 19, age < 75), but in all cases the magnitude of the treatment effects observed was smaller than that observed with donepezil. Treatment with donepezil led to statistically significant improvements on the ADAS-Cog 11 item total score, the CIBIC-Plus, and the ADCS-ADL. The results observed for the donepezil group are consistent with previous trials [27] and suggest that the design of the study and its conduct were sufficient to achieve assay sensitivity for these measures.

The discrepant efficacy results for the ABT-126 75 mg dose observed on the cognition measure and the secondary measures were unexpected. These results do not appear to be due to lack of assay sensitivity on the ADAS-Cog, since the donepezil group demonstrated an improvement consistent with previous trials, and suggests that the results for the ADCS-ADL and CIBIC-Plus do not constitute true signals of efficacy for ABT-126.

No serious safety signals were identified for ABT-126 monotherapy in subjects with mild-to-moderate AD in either the double-blind or extension studies. The overall rates of AEs and SAEs were similar across treatment groups in the double-blind study and led to few subjects discontinuing from the study. There were no consistent clinically meaningful changes in laboratory, vital sign, or ECG values in either study.

Constipation was the only AE that occurred in a statistically significant higher percentage of subjects treated with ABT-126 compared with placebo in the double-blind study (*P* = 0.019) and was reported in 17 subjects in the open-label study. Compared with placebo, a statistically significantly larger proportion of subjects treated with ABT-126 50 mg had gastrointestinal disorders considered to have a reasonable possibility of being related to the study drug (*P* = 0.033). This finding was likely influenced by AEs of constipation. The emergence of constipation as the most frequent AE in the double-blind study, occurring at an incidence that was statistically higher than placebo, is consistent with information reported by Forum Pharmaceuticals for their α7 agonist, encenicline, in mild-to-moderate AD [9]. Because of structural similarities between the α7 nAChR subunit and the 5HT-3 receptor, α7 agonists can bind to the 5-HT3 receptor [6, 28, 29]. ABT-126 shows a 10-fold higher affinity for α7 nAChR relative to the 5-HT3 receptor, whereas encenicline has approximately equal affinity for each receptor type, raising the possibility that off-target binding to the 5-HT3 receptor binding leads to constipation.

This study utilized a novel study design that consisted of an adaptive randomization to efficiently explore the dose–response relationship across three doses of ABT-126 compared with placebo and donepezil (Part 1) and permitted additional exploration of the efficacy and safety of the selected dose compared with placebo (Part 2). If the study had been powered to evaluate each dose arm for both primary and secondary measures in a traditional parallel-group design, the sample size would have increased to at least 100 subjects per arm for a total of 500 subjects. The sample size savings in Part 1 of the trial with adaptive randomization are related to the slope of the dose–response curve, with steeper slopes resulting in differential allocation to the selected dose and shallower slopes resulting in allocation that is roughly proportionate across dose groups. In this trial, the dose–response relationship exhibited by ABT-126 did not result in disproportionate sample size allocation; thus, the potential sample size savings based on this feature were not recognized. In simulations of other types of dose–response curves, such as no effect or an effect with ABT-126 75 mg that was much greater than placebo or the active control, a sample size saving of 227 or 150, respectively, could have been realized for the entire study compared with the parallel design with 500 subjects. In addition, had the decision been made not to proceed to Part 2 of the study, the total sample size could have been further reduced by 88 subjects. To our knowledge, this study is the first adaptive randomization design to include comparisons with both a placebo and an active control. Inclusion of an active control can be especially helpful for decision-making, because it allows the futility and success algorithms to stipulate decisions relative to each.

The decision to include adaptive design elements in a clinical trial needs to be made both with an evaluation of the potential benefits and an appreciation for the added complexity inherent in these trials. Designing the randomization scheme and implementing the Interactive Voice Response System takes longer for an adaptive trial than for conventional trial designs. Ongoing data cleaning, frequent interim evaluations and DMC meetings, and updates to the Interactive Voice Response System are some of the required activities. Overall, adaptive randomization is most valuable in dose–response studies with a high number of dose arms. Potential advantages (such as a lower sample size) need to be examined on a case-by-case basis to determine suitability for a particular study.

One limitation of this study is that, consistent with clinical practice at the time of study execution, the NINCDS/ADRDA criteria were used for the diagnosis of AD in study subjects and there was no confirmation of the presence of cerebral amyloid. Based on neuropathological analysis of patients meeting diagnostic criteria for AD [30, 31] and on recent clinical trial results showing that approximately 30 % of subjects selected with these criteria have negative positron emission tomography scans for amyloid [32, 33], we expect that some subjects in this trial would also be amyloid negative and would likely not have dementia due to AD. Subjects with other causes of cognitive impairment may not progress over time, contributing to a lack of decline in the placebo group and increasing the difficulty of eliciting a treatment response. In this trial, the placebo decline on the ADAS-Cog was larger in the moderate AD subgroup, likely reflecting both a higher percentage of subjects that are amyloid positive and a greater degree of sensitivity for the ADAS-Cog to detect changes in this subgroup, and it was this subgroup that demonstrated small but statistically significant treatment effects. Subgroup analyses investigating treatment effects by MMSE score, age, gender, country, and apolipoprotein E status were limited by the number of subjects included in each subgroup.

In parallel with execution of this monotherapy trial, a second trial investigating the efficacy and safety of ABT-126 as an add-on treatment to AChEIs was also conducted. In that trial, 25 mg or 75 mg ABT-126 failed to demonstrate statistically significant improvement relative to placebo. There was some evidence in that trial for a potential treatment effect in the subjects with mild AD [34]. This contrasts with the subgroup analysis presented here for the monotherapy trial where some evidence for efficacy was observed in the moderate AD subpopulation. Taken together, these data suggest that further exploration of efficacy in either of these subgroups is not warranted.

Overall, the data from the phase 2b program for ABT-126 as monotherapy or add-on therapy in mild-to-moderate AD do not support further development of this compound. With the two phase 3 studies of the α7 nAChR partial agonist encenicline (ClinicalTrials.gov NCT01969123 and NCT01969136) being placed on clinical hold [9], the future development of other agents in this class is uncertain.

## Conclusions

ABT-126 did not result in statistically significant improvement in the primary measure of cognition at any dose. The results obtained with donepezil treatment indicate that the design and conduct of the study were sufficient to detect a meaningful treatment difference. Significant improvement in secondary measures of daily function and global improvement were seen with ABT-126 50 mg and ABT-126 75 mg, but the magnitude of the effect did not support further development of ABT-126 for the monotherapy treatment of mild-to-moderate AD. Overall there were no serious safety signals identified for ABT-126 in subjects with AD.

## Additional files

Additional file 1: Presents a list of institutional review boards and independent ethics committees. (DOCX 26 kb)
Additional file 2: Presents adaptive randomization information. (DOCX 68 kb)
Additional file 3: Is a table presenting the timing of secondary efficacy assessments in the double-blind study. (DOCX 28 kb)
Additional file 4: Presents subgroup analyses and results. (DOCX 31 kb)

## Abbreviations

AChEI: Acetylcholinesterase inhibitor
AD: Alzheimer’s dementia
ADAS-Cog: Alzheimer’s Disease Assessment Scale—Cognitive subscale
ADCS-ADL: Alzheimer’s Disease Cooperative Study—Activities of Daily Living
AE: Adverse event
ANCOVA: Analysis of covariance
APOE: Apolipoprotein E
CIBIC: Clinician Interview-Based Impression of Change
CIBIS: Clinician Interview-Based Impression of Severity
CSDD: Cornell Scale for Depression in Dementia
C-SSRS: Columbia-Suicide Severity Rating Scale
DEMQOL: DEMentia Quality of Life
DMC: Data Monitoring Committee
ECG: Electrocardiogram
EQ-5D-3 L: EuroQol-5D-3 L
EQ-5D-5 L: EuroQol-5D-5 L
ITT: Intent-to-treat
LS: Least squares
NINCDS/ADRDA: National Institute of Neurological and Communicative Disorders and Stroke/Alzheimer’s Disease and Related Disorders Association
MHIS: Modified Hachinski Ischemic Scale
MMRM: Mixed-effects model for repeated measures
MMSE: Mini-Mental Status Examination
nAChR: Nicotinic acetylcholine receptor
NPI: Neuropsychiatric Inventory
PPQSA: Partner–Patient Questionnaire for Shared Activities
QD: Once daily
RUD-Lite: Resource Use in Dementia
SAE: Serious adverse event
WMS-III: Wechsler Memory Scale—III
