# Supplementary material for: ABT-126 monotherapy in mild-to-moderate Alzheimer’s dementia: randomized double-blind, placebo and active controlled adaptive trial and open-label extension
Source: Alzheimers Res Ther. 2016 Oct 18;8:44. doi: 10.1186/s13195-016-0210-1 (PMC5067903; doi:10.1186/s13195-016-0210-1)
Supplement: Additional file 1: — Presents a list of institutional review boards and independent ethics committees. (DOCX 26 kb) [file 13195_2016_210_MOESM1_ESM.docx]

**Additional File 4: List of Institutional Review Boards and Independent Ethics Committees**

Komisja Bioetyczna OIL

ul. M. Sklodowskiej-Curie 11

Szczecin, 71-332

Poland

City Clinical Hospital #61

Ethics Committee of State Institution of Healthcare of Moscow

15 Dovatora street

Moscow, 119048

Russian Federation

City Neurological Center Sibneuromed LLC

Local Ethics Committee

Michurina street 37

Novosibirsk, 630091

Russian Federation

Ethics Counsel under the Ministry of Healthcare

3, Rakhmanovskiy pereulok

Moscow, 127994

Russian Federation

Federal State Institution Independent Ethics Committee

Saint-Petersburg Scientific Research

Psychoneurological Institute named after V.M. Bekhterev of

Ministry of Health and Social

Development of the Russian

St. Petersburg, 192019

Russian Federation

Kazan State Medical University Local Ethics Committee

Butlerova Street 49

Kazan, TA 420012

Russian Federation

Kirov City Clinical Hospital 1

Local Ethics Committee

41, Popov street

Kirov, 610014

Russian Federation

Ltd Liability Co Scientific Res Med Complex

Your Health Ethics Committee

7, Zinina street

Kazan, TA 420097

Russian Federation

Saint Sofia Saratov Regional Psychiatric Hospital

LEC of State Healthcare Institution

Steinberg S.I. Str. 50

Saratov, 410060

Russian Federation

St. Petersburg Geriatric Medical and Social Center

Ethic Committee of Saint-Petersburg

State Health Care Institution

148, nab. reki Fontanki

St. Petersburg, 190103

Russian Federation

State Budgetary Educational Institution

Medical Ethics Council of experts of Russian medical academy postgraduate education

Bld. 1, 2/1 Barrikadnaya Street

Moscow, 123995

Russian Federation

Institute of Aging in Africa, Univ Cape Town

Groote Schuur Hospital, Human

Research Ethics Committee

Faculty Health Sciences, Rm E52-24,

Old Main Bldg, Observatory

Cape Town, WC 7925

South Africa

University of the Witwatersrand Human Research EC, Medical

2nd floor

8 Blackwood Avenue, Parktown

Johannesburg, GT 2193

South Africa

Donetsk National Medical University named after M Gorky

Local Ethics Commission of Communal medical and Preventive Treatment

Institution Regional Clinical Psychiatric Hospital

19, Odinstsova Str.

Donetsk, 83037

Ukraine

Kiev City Clinical Hospital No. 9

Communal Institution, LEC

1, Ryzka Strreet

Kiev, 04112

Ukraine

Poltava Regional Clinical Psychiatric Hospital named after O.F. Maltsev

1, Medychna Street

Poltava, 36006

Ukraine

NRES Committee South Central –Southampton A

South West REC Centre

Level 3, Block B, Whitefriars

Lewins Mead

Bristol, BS1 2NT

United Kingdom

Quorum Review IRB

Suite 1000

1601 Fifth Avenue

Seattle, WA 98101

United States
