# Supplementary material for: ABT-126 monotherapy in mild-to-moderate Alzheimer’s dementia: randomized double-blind, placebo and active controlled adaptive trial and open-label extension
Source: Alzheimers Res Ther. 2016 Oct 18;8:44. doi: 10.1186/s13195-016-0210-1 (PMC5067903; doi:10.1186/s13195-016-0210-1)
Supplement: Additional file 2: — Presents adaptive randomization information. (DOCX 68 kb) [file 13195_2016_210_MOESM2_ESM.docx]

**Additional File 1: Adaptive Randomization**

**Methods**

The algorithm for creating adaptive randomization probabilities was based on the probability that each arm is the maximum effective dose (ED_max_) of ABT-126 and assigned more subjects to the dose with a high probability of being an ED_max_ at each interim efficacy evaluation (Figure 1). The updated randomization probability for each ABT-126 treatment group was generated at 2-week intervals utilizing all available data at different visits for all randomized subjects. Change from baseline on 11-item ADAS-Cog total scores were used to calculate the posterior probability distributions for mean from baseline change parameters with a dose-response relationship assumed to be a Normal Dynamic Linear Model. The estimated dose-response information was used to update the randomization ratio such that the likelihood of assigning a subject to a particular dose of ABT-126 was proportional to the probability of that dose being ED_max_. The randomization ratio to placebo and donepezil group was fixed at 0.20 for Part 1.

The ADAS-Cog was assessed at screening visits 1 and 2, day –1 (baseline) and at weeks 4, 8, 12, 18 and 24. For the interim evaluations a longitudinal model was developed from available data to impute week 24 scores from subjects who had not reached week 24 to allow the adaptive algorithm to use as much available information as possible. The longitudinal model was updated dynamically from available data at early weeks to impute not yet observed week 24 ADAS-Cog scores, which were then included into the adaptation algorithm. When week 24 observations became available these observations were used in the adaptation algorithm. The use of Bayesian longitudinal modeling maximized the amount of information included for adapting the randomization allocation ratio and enhanced the precision of estimating the dose-response relationships.

After 150 subjects were randomized, the efficacy data monitoring committee (DMC) further assessed whether pre-specified criteria for stopping the study were met and made recommendations to the study sponsor regarding whether subject enrollment should continue, stop due to futility, or stop Part 1 due to success and initiate Part 2. Part 1 of the study could stop early for success if the predictive probability of phase 3 success for the most likely ED_max_ was at least 0.9 and the posterior probability that the mean improvement in the ADAS-Cog of the most likely ED_max_ was better than donepezil by at least 0.85. The study could stop early for futility if the predictive probability of phase 3 success for the most likely ED_max_ was less than 0.2, or the posterior probability that the mean improvement in the ADAS-Cog of the most likely ED_max_ was better than donepezil was less than 0.1.

The maximum sample size for Part 1 was 350 subjects. If Part 1 was not stopped due to futility or safety concerns, the efficacy DMC would 1) determine whether the study proceeded to Part 2 when 330 subjects were randomized and 2) recommend which ABT-126 dose would be used in Part 2 based on the accumulated efficacy and safety data at that point.

**Results**

Interim adaptation of randomization began after 100 subjects had been randomized. Randomization probabilities were updated every 2 weeks based on cumulative efficacy information throughout the study. At the final analysis in Part 1, subjects were distributed across treatment dose groups as follows: 60 placebo, 77 ABT-126 25 mg, 64 ABT 126 50 mg, 73 ABT-126 75 mg, 76 donepezil. After review of the interim efficacy results when 330 subjects were enrolled, it was decided that the trial would proceed to Part 1 using ABT-126 50 mg. In Part 2 88 additional subjects were enrolled, 40 to ABT-126 50 mg and 48 to placebo, resulting in a total of 104 subjects in the placebo group and 108 in the 50 mg group.

Figure 1. Adaptive randomization schematic


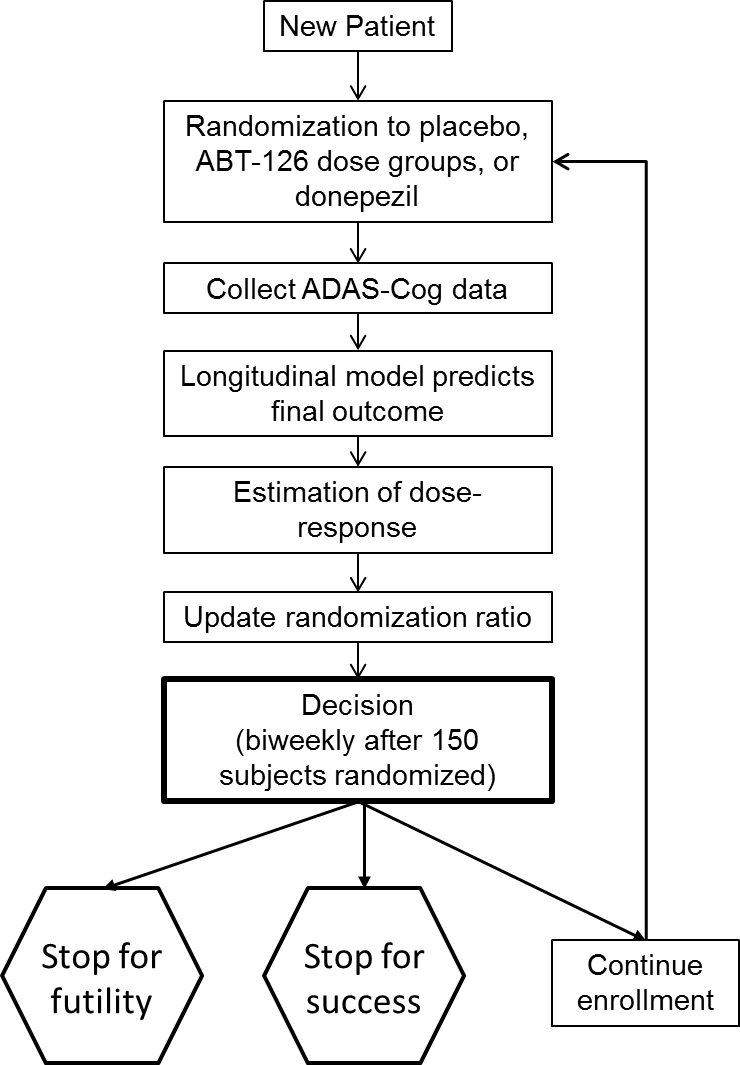


This adaptive randomization was employed in Part 1 of the randomized study after 100 subjects had been randomized.
