# Supplementary material for: ABT-126 monotherapy in mild-to-moderate Alzheimer’s dementia: randomized double-blind, placebo and active controlled adaptive trial and open-label extension
Source: Alzheimers Res Ther. 2016 Oct 18;8:44. doi: 10.1186/s13195-016-0210-1 (PMC5067903; doi:10.1186/s13195-016-0210-1)
Supplement: Additional file 3: — Is a table presenting the timing of secondary efficacy assessments in the double-blind study. (DOCX 28 kb) [file 13195_2016_210_MOESM3_ESM.docx]

**Additional File 2: Timing of Secondary Efficacy Assessments in the Double-Blind Study**

|  | **Screening Period** | | | **Treatment Period** | | | | | |
| --- | --- | --- | --- | --- | --- | --- | --- | --- | --- |
|  | **Screen Visit 1** | **Screen Visit 2** | **Day −1** | **Wk 2** | **Wk 4** | **Wk 8** | **Wk 12** | **Wk 18** | **Wk 24** |
| 13-item ADAS-Cog | X | X | X |  | X | X | X | X | X |
| MMSE | X | X | X |  | X | X | X | X | X |
| CIBIC-plus (CIBIS during screening) |  |  | X |  | X |  | X |  | X |
| NPI |  |  | X |  | X |  | X |  | X |
| ADCS-ADL | X |  | X |  | X |  | X |  | X |
| WMS-III  Working Memory Index |  | X |  |  |  | X |  | X |  |
| DEMQOL/  DEMQOL Proxy |  |  | X |  |  |  | X |  | X |
| PPQSA |  | X |  |  |  | X |  |  | X |
| RUD-Lite |  |  | X |  |  |  |  |  | X |
| EQ-5D-5L |  |  | X |  |  |  |  |  | X |
| EQ-5D-3L Proxy |  |  | X |  |  |  |  |  | X |

ADCS-ADL=Alzheimer's Disease Cooperative Study – Activities of Daily Living; ADAS-Cog=Alzheimer’s disease Assessment Scale-Cognitive subscale; CIBIC=Clinician Interview-Based Impression of Change; CIBIS=Clinician Interview-Based Impression of Severity;DEMQOL=DEMentia Quality of Life; EQ-5D-3L=EuroQol-5D-3L; EQ-5D-5L=EuroQol-5D-5L; MMSE=Mini Mental Status Examination; NPI=Neuropsychiatric Inventory; PPQSA=Partner-Patient Questionnaire for Shared Activities; RUD-Lite=Resource Use in Dementia; Wk=week; WMS-III=Wechsler Memory Scale-III
