# Supplementary material for: ABT-126 monotherapy in mild-to-moderate Alzheimer’s dementia: randomized double-blind, placebo and active controlled adaptive trial and open-label extension
Source: Alzheimers Res Ther. 2016 Oct 18;8:44. doi: 10.1186/s13195-016-0210-1 (PMC5067903; doi:10.1186/s13195-016-0210-1)
Supplement: Additional file 4: — Presents subgroup analyses and results. (DOCX 31 kb) [file 13195_2016_210_MOESM4_ESM.docx]

**Additional File 3: Subgroup Analyses and Results**

Pre-specified subgroup analyses of the ADAS-Cog 11-item, ADCS-ADL, basic ADCS-ADL, and instrumental ADCS-ADL total scores were conducted using baseline MMSE score ≥ 20 or ≤ 19, gender, age < 75 or ≥ 75), apolipoprotein E (APOE) )-ε4+ or negative, and country. An analysis of covariance (ANCOVA) model with the categorical effect of treatment, study site and subgroup variable and the treatment-by-subgroup variable interaction was utilized and the corresponding baseline score was included as a covariate. Subgroup analyses of the NPI 10-item total score were also evaluated using the same ANCOVA model with baseline NPI 10-item total score ≥ 10 or < 10 and baseline apathy score ≥ 4 or < 4 as variables.

Key findings are summarized in Table 1. Statistically significant treatment-by-subgroup variable interactions on the ADAS-Cog 11-item total score were observed for baseline MMSE category, (*P* = 0.006), age (*P* = 0.041), and country (*P* = 0.005). In subjects with moderate disease severity (MMSE values ≤ 19 at baseline), improvements vs. placebo were seen for ABT-126 50 mg (*P* = 0.031), ABT-126 75 mg (*P* = 0.024), and donepezil (*P* < 0.001) but no significant changes in any treatment group were seen in subjects with baseline MMSE scores ≥ 20. Subjects < 75 years had significant improvement in the ABT-126 75 mg group (*P* = 0.041) compared with placebo. Significant improvements in were seen in subjects from the Ukraine treated with ABT-126 50 mg (*P* = 0.008), ABT-126 75 mg (*P* < 0.001), and donepezil (*P* < 0.001). No treatment-by-subgroup variable interaction was observed for gender or APOE status.

A statistically significant treatment-by-subgroup variable interaction was observed on the ADCS-ADL, basic ADCS-ADL, and instrumental ADCS-ADL total scores for baseline MMSE category, where subjects with MMSE ≤ 19 had significant improvements in all treatment groups (all *P* < 0.05). In addition, subjects treated with ABT-126 50 mg < 75 years had significant improvement for the basic ADCS-ADL (*P* = 0.036). No other treatment-by-subgroup interactions were seen for country, age, or APOE status and no interactions were seen for the NPI 10-item total score.

**Table 1. Summary of Statistically Significant ANCOVA Subgroup Analyses**

|  |  | **Baseline** | **Change from baseline to final** | | |
| --- | --- | --- | --- | --- | --- |
| **Scale: Subgroup** | **n** | **Mean (SD)** | **Mean (SD)** | **LS Mean (SE)** | **P-value** |
| **ADAS-Cog 11-item: MMSE ≤19** | | | | | |
| Placebo | 49 | 33.9 (9.97) | 1.2 (5.66) | 2.6 (0.87) | -- |
| ABT-126 25 mg | 28 | 32.8 (10.36) | -0.0 (6.46) | 0.6 (1.09) | 0.066 |
| ABT-126 50 mg | 57 | 30.8 (9.18) | 0.0 (6.92) | 0.6 (0.77) | 0.031* |
| ABT-126 75 mg | 38 | 32.4 (9.27) | -0.4 (6.09) | 0.2 (0.96) | 0.024* |
| Donepezil | 35 | 35.9 (8.10) | -4.0 (5.82) | -2.0 (1.03) | <0.001 |
| **ADAS-Cog 11-item: age < 75 years** | | | | | |
| Placebo | 54 | 26.7 (11.19) | -0.2 (5.51) | 0.0 (0.78) | -- |
| ABT-126 25 mg | 36 | 22.7 (11.13) | 0.2 (5.89) | 0.8 (0.97) | 0.748 |
| ABT-126 50 mg | 47 | 24.0 (11.17) | -1.6 (6.25) | -1.6 (0.85) | 0.077 |
| ABT-126 75 mg | 29 | 27.7 (10.23) | -2.5 (5.24) | -2.2 (1.06) | 0.41* |
| Donepezil | 29 | 29.9 (12.50) | -3.6 (5.25) | -2.0 (1.08) | 0.57 |
| **ADAS-Cog 11-item: Ukraine** | | | | | |
| Placebo | 15 | 36.0 (10.86) | 3.1( 6.13) | 3.5 (1.41) | -- |
| ABT-126 25 mg | 4 | 27.8 (10.24) | 5.8 (6.36) | -0.2 (2.94) | 0.130 |
| ABT-126 50 mg | 9 | 40.1( 8.49) | -1.8 (12.96) | -2.0 (1.84) | 0.008* |
| ABT-126 75 mg | 6 | 31.7( 9.01) | -6.2 (8.07) | -6.6 (2.21) | <0.001* |
| Donepezil | 13 | 36.6( 9.45) | -8.3 (6.79) | -7.4 (1.57) | <0.001* |
| **ADCS-ADL Total Score: MMSE ≤19** | | | | | |
| Placebo | 49 | 49.0 (14.81) | -4.3 (8.72) | -6.4 (1.08) | -- |
| ABT-126 25 mg | 28 | 49.1 (17.46) | -0.9 (5.61) | -2.3 (1.38) | 0.008* |
| ABT-126 50 mg | 57 | 49.8 (15.79) | -0.9 ( 7.87) | -1.8( 0.98) | <0.001* |
| ABT-126 75 mg | 38 | 51.8 (15.47) | -1.3 (7.72) | -1.6 (1.21) | 0.001* |
| Donepezil | 36 | 45.4 (13.99) | 3.7( 6.12) | 1.1 (1.28) | <0.001* |
| **Basic ADCS-ADL: MMSE ≤19** | | | | | |
| Placebo | 49 | 18.4 (3.77) | -1.3 (2.92) | -1.8 (0.33) | -- |
| ABT-126 25 mg | 28 | 18.3 (4.69) | -0.2 (1.68) | -0.4 (0.42) | 0.005* |
| ABT-126 50 mg | 57 | 18.2 (4.17) | -0.1 (2.64) | -0.4 (0.30) | <0.001* |
| ABT-126 75 mg | 38 | 19.2 (3.46) | 0.1 (1.96) | -0.1 (0.37) | <0.001* |
| Donepezil | 36 | 18.0 (3.52) | 0.4 (2.80) | -0.3 (0.39) | <0.001* |
| **Basic ADCS-ADL: Age < 75 years** | | | | | |
| Placebo | 54 | 19.5 (3.25) | -0.4 (1.86) | -0.5 (0.30) | **--** |
| ABT-126 25 mg | 36 | 20.1 (2.98) | -0.1 (1.41) | -0.1 (0.38) | 0.256 |
| ABT-126 50 mg | 47 | 19.9 (3.44) | 0.4 (1.44) | 0.3 (0.33) | 0.036* |
| ABT-126 75 mg | 29 | 18.6 (3.49) | 0.0 (1.09) | 0.2 (0.41) | 0.090 |
| Donepezil | 30 | 18.7 (2.86) | -0.2 (2.36) | -0.6 (0.41) | 0.640 |
| **Instrumental ADCS-ADL: MMSE ≤19** | | | | | |
| Placebo | 49 | 30.7 (11.68) | -3.0 (6.82) | -4.8 (0.88) | -- |
| ABT-126 25 mg | 28 | 30.9 (13.32) | -0.7 (4.95) | -2.1 (1.13) | 0.026* |
| ABT-126 50 mg | 57 | 31.6 (12.44) | -0.8 (6.26) | -1.6 (0.80) | 0.002* |
| ABT-126 75 mg | 38 | 32.7 (12.74) | -1.3 (6.73) | -1.6 (0.99) | 0.007* |
| Donepezil | 36 | 27.4 (11.18) | 3.3 (4.38) | 1.2 (1.04) | <0.001* |

Abbreviations: ADCS-ADL = Alzheimer’s Disease Cooperative Study-Activity of Daily Living; ADAS-Cog = Alzheimer’s Disease Assessment Scale-cognitive subscale; ANCOVA = analysis of covariance; MMSE = Mini-mental Status Examination; LS = least squares; n = number of subjects; SD = standard deviation; SE = standard error

*One-sided p-value < 0.05
